# Prognostic value of initial and longitudinal changes in body composition in metastatic pancreatic cancer

**DOI:** 10.1002/jcsm.13437

**Published:** 2024-02-08

**Authors:** Min Woo Lee, Sun Kyung Jeon, Woo Hyun Paik, Jeong Hee Yoon, Ijin Joo, Jeong Min Lee, Sang Hyub Lee

**Affiliations:** ^1^ Department of Internal Medicine and Liver Research Institute Seoul National University Hospital, Seoul National University College of Medicine Seoul South Korea; ^2^ Department of Internal Medicine Armed Forces Capital Hospital Seongnam South Korea; ^3^ Department of Radiology Seoul National University Hospital Seoul South Korea; ^4^ Institute of Radiation Medicine Seoul National University Medical Research Center Seoul South Korea

**Keywords:** body composition analysis, deep learning, pancreatic cancer, survival

## Abstract

**Background:**

Sarcopenia or visceral adipose tissue has been reported to be related to pancreatic cancer prognosis. However, clinical relevance of the comprehensive analysis of body compositions and their longitudinal changes is lacking. This study analysed the association between body composition changes after chemotherapy and survival in patients with metastatic pancreatic cancer.

**Methods:**

We retrospectively included 456 patients (mean age ± standard deviation, 61.2 ± 10.0 years; 272 males and 184 females) with metastatic pancreatic cancer who received palliative chemotherapy from May 2011 to December 2019. Using deep learning‐based, fully automated segmentation of contrast‐enhanced computed tomography (CT) at the time of diagnosis, cross‐sectional areas of muscle, subcutaneous adipose tissue and visceral adipose tissue were extracted from a single axial image of the portal venous phase at L3 level. Skeletal muscle index (SMI), visceral adipose tissue index (VATI), subcutaneous adipose tissue index (SATI) and mean skeletal muscle attenuation (MA) were calculated, and their effect on overall survival (OS) was analysed. Longitudinal changes in body composition and prognostic values were also analysed in a subgroup of patients with 2‐ and 6‐month follow‐up CT (*n* = 349).

**Results:**

A total of 452 deaths occurred during follow‐up in the entire cohort. The survival rate was 49.3% (95% confidence interval [CI], 44.9–54.2) at 1 year and 3.7% (95% CI, 2.0–6.8) at 5 years. In multivariable analysis, higher MA (≥44.4 HU in males and ≥34.8 HU in females) at initial CT was significantly associated with better OS in both males and females (adjusted hazard ratio [HR], 0.706; 95% CI, 0.538–0.925; *P* = 0.012 for males, and HR, 0.656; 95% CI, 0.475–0.906; *P* = 0.010 for females), whereas higher SATI (≥42.8 cm^2^/m^2^ in males and ≥65.8 cm^2^/m^2^ in females) was significantly associated with better OS in female patients only (adjusted HR, 0.568; 95% CI, 0.388–0.830; *P* = 0.003). In longitudinal analysis, SMI, VATI and SATI significantly decreased between initial and 2‐month follow‐up CT, whereas mean MA significantly decreased between 2‐ and 6‐month follow‐up CT. In multivariable Cox regression analysis of longitudinal changes, which was stratified by disease control state, SATI change was significantly associated with OS in male patients (adjusted HR, 0.513; 95% CI, 0.354–0.745; *P* < 0.001), while other body composition parameters were not.

**Conclusions:**

In patients with metastatic pancreatic cancer, body composition mostly changed during the first 2 months after starting chemotherapy, and the prognostic factors associated with OS differed between males and females. Initial and longitudinal changes of body composition are associated with OS of metastatic pancreatic cancer.

## Introduction

Pancreatic cancer is one of the most lethal cancers and the seventh leading cause of cancer‐related death in both sexes worldwide.^1^ This is because 80–85% of patients are diagnosed in an advanced stage, even though the only curative option is surgical resection.^2^, ^3^ Despite recent developments in systemic therapy, the 5‐year survival rate of patients with metastatic pancreatic cancer remains <5%.^2^ In addition to tumour stage and tumour biology, including tumour marker levels, metabolic factors such as sarcopenia and body composition are also major prognostic factors for pancreatic cancer.^4^, ^5^ Several studies have demonstrated the association between sarcopenia and surgical outcome in localized pancreatic cancer.^6^, ^7^ Although considerable research has been devoted to sarcopenia in localized pancreatic cancer, relatively less attention has been paid to other body compositions and metastatic pancreatic cancer.

Computed tomography (CT) is a non‐invasive imaging method for analysing body composition, including skeletal muscle and visceral and subcutaneous adipose tissues.^8^ The mass of each body compartment can be evaluated by quantitative measurements of segmented area of skeletal muscle, subcutaneous adipose tissue and visceral adipose tissue. In addition, skeletal muscle radiation attenuation, which is associated with lipid content of muscle, can be used in body composition analysis.^9^ With the recent developments in deep learning techniques, body composition analysis using CT segmentation has become more accurate and completely automated.^10^, ^11^ There have been reports that a deep learning‐based fully automated CT segmentation technique could be helpful in predicting outcomes in several malignancies including pancreatic cancer, and it has the potential to be incorporated into clinical practice.^12^, ^13^, ^14^ However, effects of longitudinal changes in body compositions on the prognosis of patients with pancreatic cancer who are receiving chemotherapy have not yet been thoroughly investigated.

Therefore, this study aimed to investigate the association of body composition and longitudinal changes after chemotherapy, measured using a deep learning‐based fully automated CT segmentation technique, with metastatic pancreatic cancer prognosis.

## Methods

This study was approved by the Institutional Review Board (IRB) of Seoul National University Hospital, which waived the requirement of informed consent because of the retrospective study design (IRB No. H‐2102‐058‐1195). This study was a retrospective cohort study and was reported in accordance with the STROBE guideline (*Data S1*).

### Study population

Using our electronic medical record system, we identified consecutive patients who were histologically diagnosed with pancreatic ductal adenocarcinoma with distant metastasis at our academic medical centre between May 2011 and December 2019.

The following patients were included: (1) patients with histologically confirmed metastatic pancreatic ductal adenocarcinoma by endoscopic ultrasound‐guided biopsy, (2) patients who underwent palliative chemotherapy (FOLFIRINOX or gemcitabine and nano‐albumin‐bounded paclitaxel) and (3) patients who had an initial (baseline) contrast‐enhanced abdominal CT before starting the chemotherapy. The following patients were excluded: (1) those without initial CT scan within 1 month before starting chemotherapy and (2) when initial CT did not include enhanced phase.

Clinical data, including patient demographics, pretreatment height, pretreatment weight, pretreatment body mass index (BMI), presence of diabetes or dyslipidaemia, carbohydrate antigen 19‐9 (CA 19‐9) level, presence of biliary obstruction or duodenal obstruction, Eastern Cooperative Oncology Group performance score and the Charlson comorbidity index, were thoroughly investigated during hospitalization for endoscopic ultrasound‐guided biopsy. Underlying diseases such as diabetes and dyslipidaemia were evaluated based on past diagnosis record and prescribed medications. The initial tumour size and location, location of distant metastasis and presence of peritoneal seeding were also collected. Peritoneal seeding was determined by the presence of seeding nodules with ascites on enhanced CT scan or by the presence of seeding nodules with visible uptake on positron emission tomography (PET) scan. CT protocol used in this study is summarized in *Document S1*.

### Computed tomography‐based body composition analysis

Portal venous phase images of anonymized initial (at the time of diagnosis), 2‐month and 6‐month follow‐up CT were uploaded to commercially available deep learning‐based software for whole‐body composition analysis (DeepCatch v1.0.0.0; MEDICALIP Co. Ltd., Seoul, South Korea). The software provides automatic volumetric segmentation of body components into the following seven classes, with an average segmentation accuracy of 97% compared with manual segmentation^15^: skin, muscle, abdominal visceral adipose tissue, subcutaneous adipose tissue, bone, internal organ, vessels and the central nervous system. Additionally, the software also provides automatic localization of the mid‐level of the L3 vertebra and automatically quantifies the L3 cross‐sectional area (square centimetres) of muscle, visceral adipose tissue and subcutaneous adipose tissue (*Figure* *S1*). Segmented muscle areas included psoas, paraspinal, transversus abdominis, rectus abdominis, quadratus lumborum, internal oblique and external oblique muscles. One abdominal radiologist (**, with 9 years of experience in body CT interpretation) who was blinded to the clinical information confirmed the appropriateness of the automatic segmentations of body composition in all cases. In 17 cases (3.7%), additional manual correction of segmentation was performed. Height‐normalized indices were computed by dividing the L3 cross‐sectional area (areas of muscle, subcutaneous adipose tissue and visceral adipose tissue) by the square of the height. These calculations yielded three distinct indices: skeletal muscle index (SMI), subcutaneous adipose tissue index (SATI) and visceral adipose tissue index (VATI). Mean radiation attenuation (in HU) of muscle area (muscle attenuation [MA]) was automatically measured by the software. For patients with 2‐ or 6‐month follow‐up CT, the interval change of SMI, SATI, VATI and MA was also evaluated. The rate of interval change was calculated using the equations below:

$$\text{δSMI}=\frac{\text{post}\;\mathrm{SMI}-\mathrm{pre}\;\mathrm{SMI}}{\mathrm{pre}\;\mathrm{SMI}}$$

$$\text{δSATI}=\frac{\text{post SATI}-\mathrm{pre}\;\text{SATI}}{\mathrm{pre}\;\text{SATI}}$$

$$\text{δVATI}=\frac{\text{post VATI}-\mathrm{pre}\;\text{VATI}}{\mathrm{pre}\;\text{VATI}}$$

$$\mathrm{\delta MA}=\frac{\text{post}\;\mathrm{MA}-\mathrm{pre}\;\mathrm{MA}}{\mathrm{pre}\;\mathrm{MA}}$$

### Outcome analysis

The primary outcome was the association between initial body composition and overall survival (OS). The longitudinal changes of body composition in the first 2 months and their effects on survival were analysed in patients with initial and 2‐month CT scan. Survival analysis was performed according to sex as the body composition of each sex revealed different distributions. The OS was defined as the duration between each time point to death. Clinical factors associated with OS, such as conversion surgery or response to chemotherapy, were predetermined and included in the survival analysis. Most factors were based on the date of the initial CT except biliary obstruction, duodenal obstruction and CA 19‐9 level. As these factors changed between time points, they were determined based on each time point.

### Statistical analysis

The difference between male and female patients was assessed using Student's *t*‐test for continuous variables and the *χ* ^2^ test and Fisher's exact test for categorical variables. Longitudinal changes of body composition indices were analysed using Friedman's test followed by Nemenyi's test as a post hoc analysis. Survival analyses were divided into two stages: initial and 2 months. Survival analysis of the initial time point included clinical factors and body composition indices. The standard value of each body composition index according to sex was calculated with maximally selected rank statistics, as in other studies.^4^, ^16^, ^17^ Survival analysis of initial time points was presented using Kaplan–Meier curves and the log‐rank test. Whether the patient underwent conversion surgery was considered as a time‐varying covariate. In the survival analysis of the 2‐month time point, the Cox regression model was stratified by disease control state. Variables with a *P* value < 0.2 in the univariable analysis were considered candidates for the multivariable model, which was determined by the bi‐directional stepwise selection method. All statistical analyses were conducted with R 4.2.1 software (http://www.r‐project.org) and considered statistically significant when two‐sided *P* value is <0.05.

## Results

### Study population

**Figure 1:**
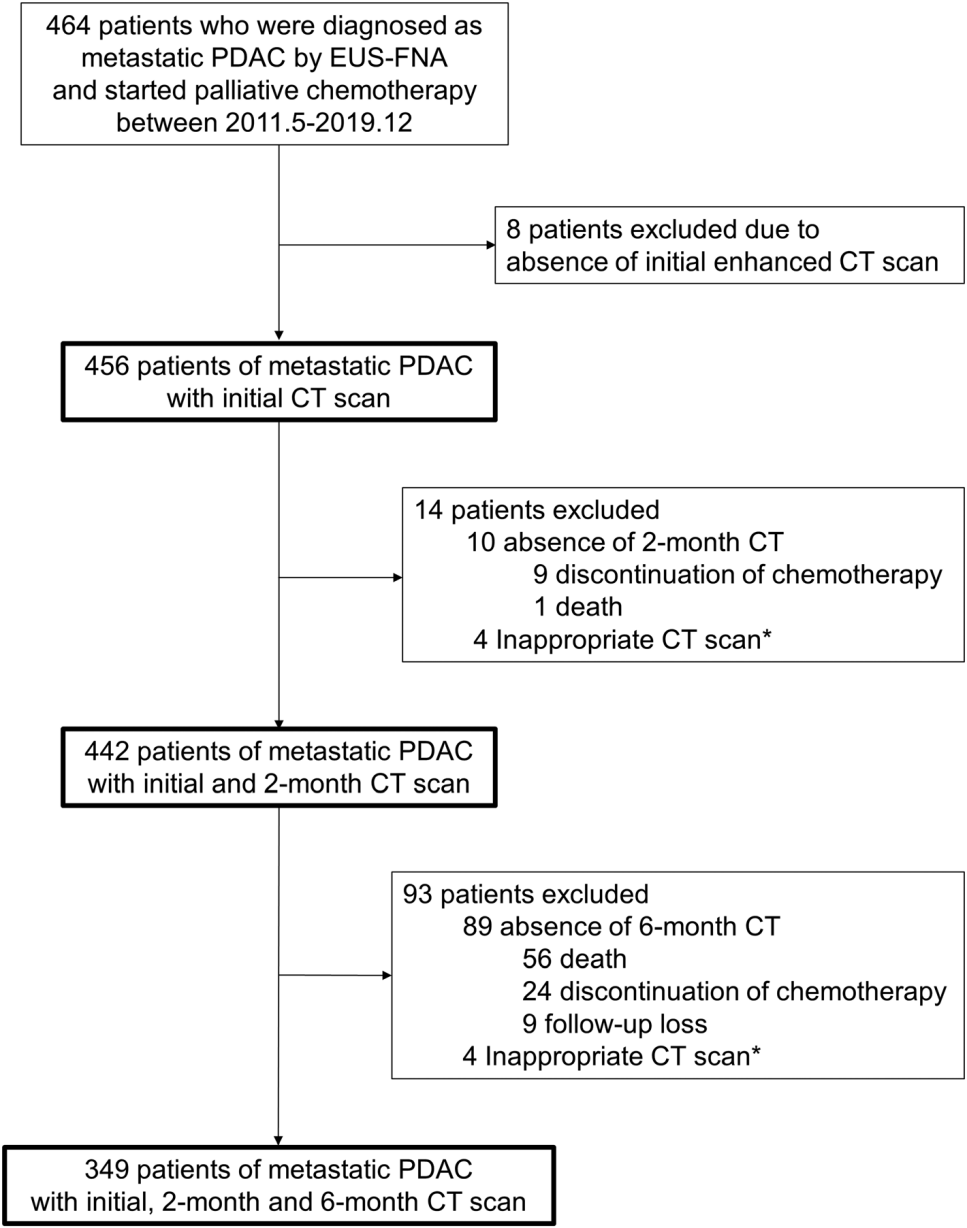
Study population. CT, computed tomography; EUS‐FNA, endoscopic ultrasound‐guided fine needle aspiration; PDAC, pancreatic ductal adenocarcinoma. *Inappropriate CT scan: Difficult to calculate visceral adipose tissue index and subcutaneous adipose tissue index due to ascites and subcutaneous oedema.

Among 464 patients diagnosed with metastatic pancreatic cancer during the study period, 8 were excluded due to the absence of initial CT before the treatment. Finally, a total of 456 patients (mean age ± standard deviation, 61.2 ± 10.0 years; 272 males and 184 females) were included in the analysis (*Figure* *1*). Among 456 patients, 322 patients received FOLFIRINOX, and the other 134 patients received gemcitabine and albumin‐bounded paclitaxel as first‐line chemotherapy. For the analysis using longitudinal changes of body compositions, 99 patients without 2‐month (*n* = 10; 9 discontinuation of chemotherapy and 1 death) or 6‐month follow‐up CT (*n* = 89; 24 discontinuation of chemotherapy, 56 deaths and 9 follow‐up loss) and 8 patients who had inappropriate CT scan were excluded. The main reasons for treatment discontinuation were deterioration of performance status and adverse events of chemotherapy such as peripheral neuropathy. Inappropriate CT scan was mainly due to subcutaneous oedema or ascites (grade 2 or higher), which interfere with measuring SATI/VATI. The baseline characteristics of patients without 2‐ or 6‐month CT scan are summarized in *Table* *S1*. There were no missing values for all variables in the remaining 349 patients.

The baseline characteristics of the study population (*n* = 456) are summarized in *Table* *1*. There was no significant difference in factors such as tumour size and location, number of metastatic organs and CA 19‐9 level between male and female patients. There were significant differences in all the body composition parameters between male and female patients (MA, 43.5 vs. 37.5 HU, *P* < 0.001; SMI, 46.5 vs. 41.5 cm^2^/m^2^, *P* < 0.001; VATI, 33.4 vs. 26.6 cm^2^/m^2^, *P* < 0.001; and SATI, 33.1 vs. 57.9 cm^2^/m^2^, *P* < 0.001).

**Table 1:** Baseline characteristics

	Male (*N* = 272)	Female (*N* = 184)	*P* value
Age	61.4 ± 10.2	61.0 ± 9.7	0.692
ECOG			0.809
0	63 (23.2%)	40 (21.7%)	
≥1	209 (76.8%)	144 (78.3%)	
Height	168.5 ± 5.9	155.9 ± 5.9	<0.001
Weight	64.4 ± 9.4	54.4 ± 8.6	<0.001
BMI	22.6 ± 2.8	22.4 ± 3.1	0.363
Type 2 diabetes	104 (38.2%)	54 (29.3#)	0.063
Dyslipidaemia	24 (8.8%)	30 (16.3%)	0.023
Charlson comorbidity index			0.512
<10	234 (86.0%)	163 (88.6%)	
≥10	38 (14.0%)	21 (11.4%)	
Tumour size	4.2 ± 2.0	3.9 ± 1.6	0.052
Tumour location			0.46
Head	88 (32.4%)	65 (35.3%)	
Body	89 (32.7%)	65 (35.3%)	
Tail	95 (34.9%)	54 (29.3%)	
Peritoneal seeding	84 (30.9%)	49 (26.6%)	0.382
CA 19‐9 (U/mL)	4157.0 ± 4966.5	4805.3 ± 5187.5	0.18
Biliary obstruction	39 (14.3%)	37 (20.1%)	0.135
Duodenal obstruction	9 (3.3%)	4 (2.2%)	0.669
MA (HU)	43.5 ± 8.0	37.5 ± 9.6	<0.001
SMI (cm^2^/m^2^)	46.5 ± 8.4	41.5 ± 7.4	<0.001
VATI (cm^2^/m^2^)	33.4 ± 22.2	26.6 ± 17.5	<0.001
SATI (cm^2^/m^2^)	33.1 ± 16.8	57.9 ± 22.7	<0.001

Abbreviations: BMI, body mass index; CA 19‐9, carbohydrate antigen 19‐9; ECOG, Eastern Cooperative Oncology Group; MA, muscle attenuation; SATI, subcutaneous adipose tissue index; SMI, skeletal muscle index; VATI, visceral adipose tissue index.

### Longitudinal changes in body composition

**Figure 2:**
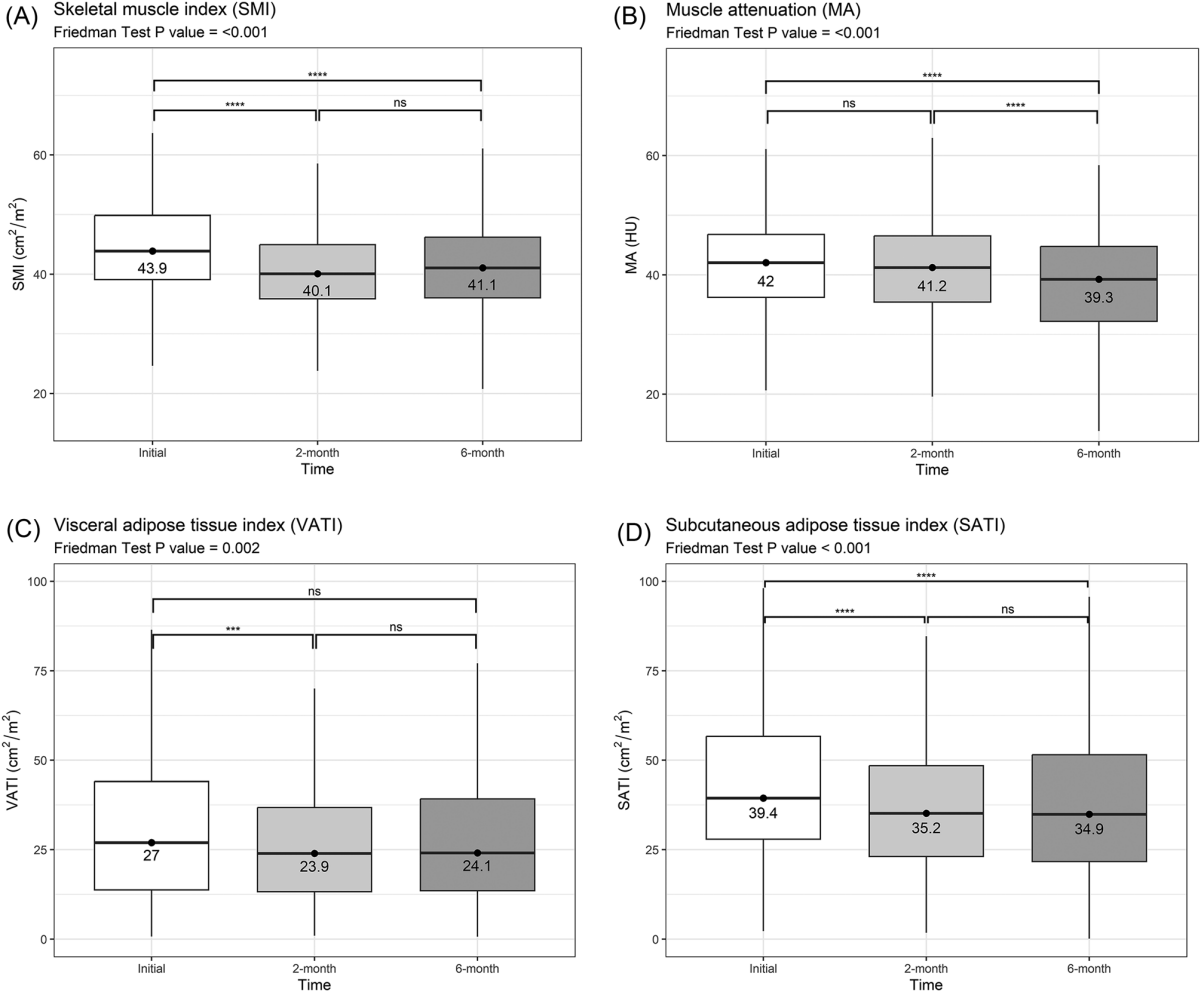
Longitudinal changes of body composition parameters. Longitudinal changes of (A) skeletal muscle index (SMI; cm^2^/m^2^), (B) muscle attenuation (MA; HU), (C) visceral adipose tissue index (VATI; cm^2^/m^2^) and (D) subcutaneous adipose tissue index (SATI; cm^2^/m^2^). SMI, VATI and SATI significantly decreased between initial and 2 months (A, C, D). Skeletal MA significantly decreased between 2 and 6 months (B). *Post hoc analysis—not significant (ns): >0.05, *: <0.05, **: <0.01, ***: <0.001, ****: <0.0001.

Longitudinal changes in body composition parameters were assessed in a subset of patients (*n* = 349) who had initial, 2‐month and 6‐month CT. MA, SMI, VATI and SATI showed significant changes in pair‐wise comparisons using Friedman's test (*Figure* *2*). In the post hoc analysis, there was a significant decrease between initial and 2‐month CT scan for SMI (median initial: 43.9 cm^2^/m^2^; interquartile range [IQR] 39.1–49.9 vs. 2‐month: 40.1 cm^2^/m^2^; IQR 35.9–45.0; *P* < 0.001), VATI (median initial: 27.0 cm^2^/m^2^; IQR 13.7–44.1 vs. 2‐month: 23.9 cm^2^/m^2^; IQR 13.2–36.8; *P* = 0.002) and SATI (median initial: 39.4 cm^2^/m^2^; IQR 27.9–56.7 vs. 2‐month: 35.2 cm^2^/m^2^; IQR 23.1–48.4; *P* < 0.001). There were no significant changes between 2 and 6 months for SMI, SATI and VATI. On the other hand, MA showed significant changes only between 2 and 6 months (median 2‐month: 41.2 HU; IQR 35.4–46.6 vs. 6‐month: 39.3 HU; IQR 32.2–44.8; *P* < 0.001).

### Survival analysis with initial body composition parameters

**Figure 3:**
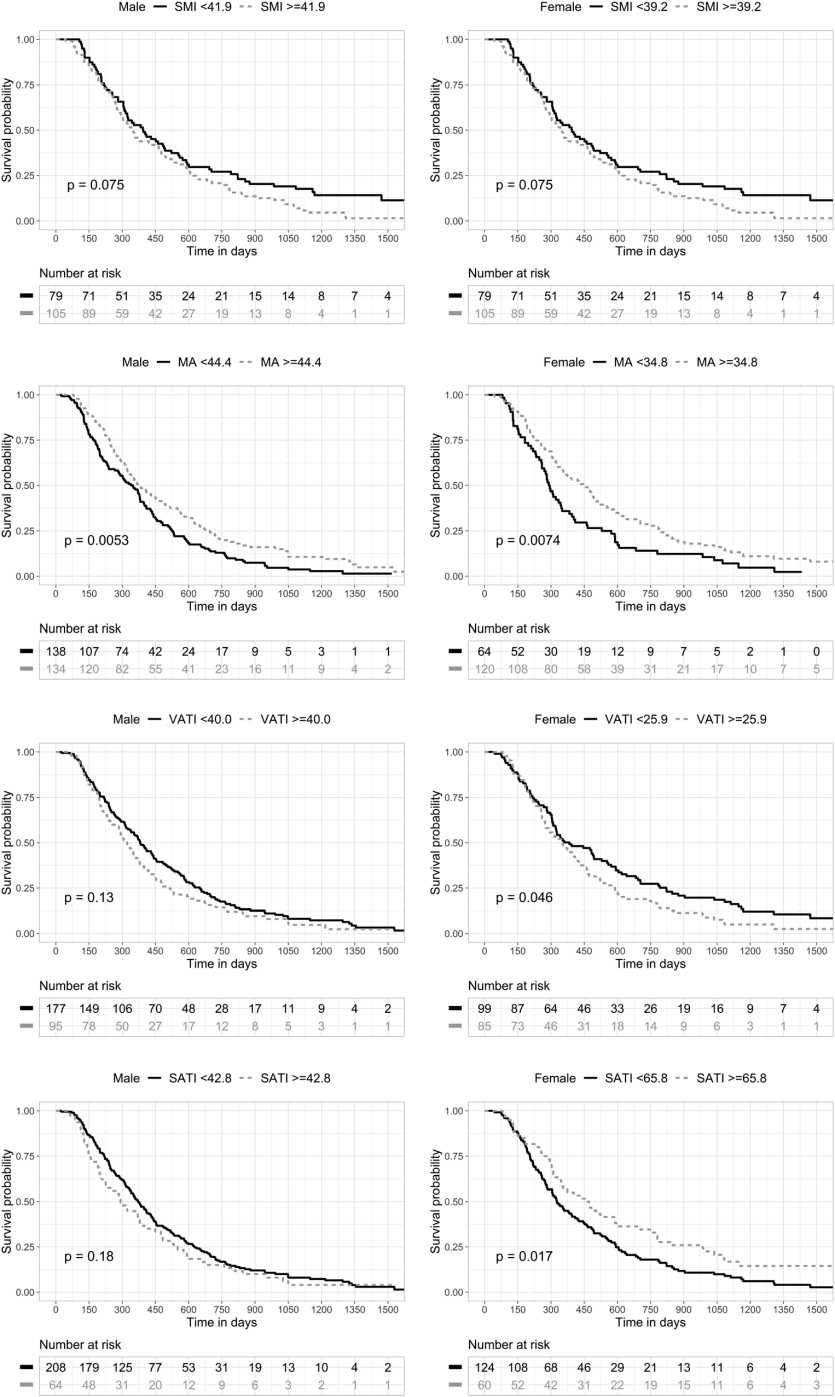
(A–D) Survival analysis with initial body composition parameters. MA, muscle attenuation; SATI, subcutaneous adipose tissue index; SMI, skeletal muscle index; VATI, visceral adipose tissue index.

Among 456 patents, there were 452 deaths during follow‐up. The median follow‐up duration was 11.7 months (IQR 6.7–20.2). The survival rate was 49.3% (95% confidence interval [CI], 44.9–54.2%) at 1 year and 9.0% (95% CI, 6.6–12.2%) and 3.7% (95% CI, 2.0–6.8%) at 5 years. To evaluate the effects of initial body composition parameters, optimal cut‐off by sex was calculated using maximally selected rank statistics (MA: 44.4 HU for males and 34.8 HU for females; SMI: 41.9 cm^2^/m^2^ for males and 39.2 cm^2^/m^2^ for females; VATI: 40.0 cm^2^/m^2^ for males and 25.9 cm^2^/m^2^ for females; and SATI: 42.8 cm^2^/m^2^ for males and 65.8 cm^2^/m^2^ for females) (*Figure* *S2*). Survival curve and log‐rank statistics of body composition parameters are shown in *Figure* *3*. Lower MA was a significant factor in poorer survival in both males and females. However, other body composition parameters were associated with OS in a different manner. Lower SMI was a significant factor only in male patients (*P* < 0.001), while higher VATI and lower SATI were significant factors only in female patients (VATI: *P* = 0.046, and SATI: *P* = 0.017). In the univariable and multivariable Cox regression analysis of male (*Table* *2*) and female patients (*Table* *3*), MA (hazard ratio [HR], 0.706; 95% CI, 0.538–0.925; *P* = 0.012 for males, and HR, 0.656; 95% CI, 0.475–0.906; *P* = 0.010 for females) and conversion surgery (HR, 0.391; 95% CI, 0.208–0.738; *P* = 0.004 for males, and HR, 0.278; 95% CI, 0.095–0.813; *P* = 0.010 for females) were significant factors in both males and females. SMI was significant in male patients (HR, 0.590; 95% CI, 0.435–0.800; *P* < 0.001), but not in female patients. SATI was significant in female patients (HR, 0.568; 95% CI, 0.388–0.830; *P* = 0.003), but not in male patients.

**Table 2:** Cox regression analysis of overall survival of male patients with initial computed tomography scan

	Univariable		Multivariable	
	HR (95% CI)	*P* value	HR (95% CI)	*P* value
Age	1.016 (1.003–1.029)	0.018		
ECOG performance score ≥ 1	1.211 (0.898–1.632)	0.209		
Type 2 diabetes	1.113 (0.861–1.439)	0.415		
Dyslipidaemia	1.080 (0.690–1.692)	0.736		
CCI ≥ 10	1.550 (1.082–2.221)	0.017	1.332 (0.902–1.968)	0.149
Size	1.058 (1.001–1.119)	0.047	1.055 (0.977–1.139)	0.172
Biliary obstruction	1.144 (0.802–1.631)	0.459		
Duodenal obstruction	0.978 (0.434–2.202)	0.957		
Peritoneal seeding	0.883 (0.673–1.159)	0.370		
CA 19‐9 ≥ 1000 U/mL	1.268 (0.985–1.632)	0.065	1.235 (0.961–1.587)	0.099
First CTx: FOLFIRINOX	0.877 (0.661–1.165)	0.365		
Conversion surgery^a^	0.369 (0.200–0.678)	0.001	0.391 (0.208–0.738)	0.004
MA ≥ 44.4 HU	0.700 (0.544–0.900)	0.005	0.706 (0.538–0.925)	0.012
SMI ≥ 41.9 cm^2^/m^2^	0.621 (0.470–0.820)	<0.001	0.590 (0.435–0.800)	<0.001
VATI ≥40.0 cm^2^/m^2^	1.226 (0.943–1.593)	0.128	1.247 (0.922–1.687)	0.152
SATI ≥ 42.8 cm^2^/m^2^	1.221 (0.911–1.637)	0.181		

Abbreviations: CA 19‐9, carbohydrate antigen 19‐9; CCI, Charlson comorbidity index; CI, confidence interval; CTx, chemotherapy; ECOG, Eastern Cooperative Oncology Group; HR, hazard ratio; MA, muscle attenuation; SATI, subcutaneous adipose tissue index; SMI, skeletal muscle index; VATI, visceral adipose tissue index.

^a^ Time‐varying covariate.

**Table 3:** Cox regression analysis of overall survival of female patients with initial computed tomography scan

	Univariable		Multivariable	
	HR (95% CI)	*P* value	HR (95% CI)	*P* value
Age	1.003 (0.987–1.020)	0.689		
ECOG ≥ 1	1.084 (0.750–1.567)	0.668		
Type 2 diabetes	0.939 (0.672–1.313)	0.714		
Dyslipidaemia	0.819 (0.535–1.255)	0.360		
CCI ≥ 10	1.438 (0.899–2.301)	0.129		
Size	1.073 (0.968–1.189)	0.181		
Biliary obstruction	1.328 (0.909–1.940)	0.143	1.401 (0.989–1.986)	0.058
Duodenal obstruction	1.598 (0.589–4.334)	0.357		
Peritoneal seeding	1.607 (1.149–2.249)	0.006	1.400 (0.989–1.986)	0.055
CA 19‐9 ≥ 1000 U/mL	1.499 (1.100–2.043)	0.010	1.539 (1.125–2.105)	0.007
First CTx: FOLFIRINOX	0.812 (0.586–1.124)	0.209		
Conversion surgery^a^	0.190 (0.061–0.598)	0.005	0.278 (0.095–0.813)	0.019
MA ≥ 34.8 HU	0.649 (0.472–0.892)	0.008	0.656 (0.475–0.906)	0.010
SMI ≥ 39.2 cm^2^/m^2^	1.327 (0.971–1.814)	0.076		
VATI ≥ 25.9 cm^2^/m^2^	1.369 (1.005–1.864)	0.046	1.331 (0.916–1.934)	0.134
SATI ≥ 65.8 cm^2^/m^2^	0.668 (0.479–0.933)	0.018	0.568 (0.388–0.830)	0.003

Abbreviations: CA 19‐9, carbohydrate antigen 19‐9; CCI, Charlson comorbidity index; CI, confidence interval; CTx, chemotherapy; ECOG, Eastern Cooperative Oncology Group; HR, hazard ratio; MA, muscle attenuation; SATI, subcutaneous adipose tissue index; SMI, skeletal muscle index; VATI, visceral adipose tissue index.

^a^ Time‐varying covariate.

### Survival analysis with longitudinal changes in body composition

Longitudinal changes of body composition parameters between initial and 2‐month CT and their effects on OS were investigated using stratified Cox regression analysis. A total of 444 patients with initial and 2‐month CT scan were included in this analysis (*Figure* *1*). Instead of including variables such as tumour size and the number of metastases, it was stratified by whether ‘progressive disease’ was identified in accordance with Response Evaluation Criteria in Solid Tumors (RECIST) 1.1 (*Table* *4*).^18^ In the multivariable model, CA 19‐9 and conversion surgery were significant prognostic factors for OS in both males and females (CA 19‐9 ≥ 1000 U/mL: HR, 1.370; 95% CI, 1.049–1.791; *P* = 0.021 for males, and HR, 1.864; 95% CI, 1.292–2.689; *P* < 0.001 for females, and conversion surgery: HR, 0.344; 95% CI, 0.176–0.672; *P* = 0.002 for males, and HR, 0.251; 95% CI, 0.076–0.821; *P* = 0.022 for females). However, δSATI was a significant prognostic factor for worse OS in males (HR, 0.531; 95% CI, 0.354–0.745; *P* < 0.001), but not in females (*P* = 0.416). Other body composition changes were not significant factors (*Figure* *S3*).

**Table 4:** Stratified Cox regression analysis of overall survival with 2‐month computed tomography scan

	Male				Female			
	Univariable		Multivariable		Univariable		Multivariable	
	HR (95% CI)	*P* value	HR (95% CI)	*P* value	HR (95% CI)	*P* value	HR (95% CI)	*P* value
Age	1.014 (1.001–1.028)	0.038	1.017 (1.003–1.032)	0.018	1.010 (0.992–1.027)	0.277		
ECOG ≥ 1	1.140 (0.842–1.542)	0.397			1.296 (0.881–1.908)	0.188		
Type 2 diabetes	1.078 (0.826–1.406)	0.580			0.928 (0.661–1.302)	0.664		
Dyslipidaemia	1.042 (0.655–1.657)	0.862			0.935 (0.606–1.441)	0.760		
CCI ≥ 10	1.297 (0.874–1.926)	0.197			1.390 (0.867–2.229)	0.171		
Biliary obstruction	1.058 (0.726–1.541)	0.770			1.110 (0.738–1.671)	0.616		
Duodenal obstruction	1.221 (0.390–3.829)	0.731			2.414 (0.759–7.681)	0.136		
Peritoneal seeding	1.037 (0.790–1.361)	0.796			1.468 (1.033–2.085)	0.032	1.323 (0.937–1.868)	0.111
CA 19‐9 ≥ 1000 U/mL	1.369 (1.055–1.776)	0.018	1.370 (1.049–1.791)	0.021	2.031 (1.463–2.819)	<0.001	1.864 (1.292–2.689)	<0.001
First CTx: FOLFIRINOX	0.797 (0.591–1.075)	0.137			0.880 (0.630–1.229)	0.454		
Conversion surgery	0.377 (0.205–0.694)	0.002	0.344 (0.176–0.672)	0.002	0.197 (0.062–0.624)	0.006	0.251 (0.076–0.821)	0.022
δMA	1.115 (0.497–2.498)	0.792			0.701 (0.307–1.599)	0.399		
δSMI	1.467 (0.674–3.193)	0.335			0.079 (0.018–0.352)	<0.001	0.171 (0.025–1.165)	0.071
δVATI	0.987 (0.771–1.265)	0.920			0.701 (0.530–0.929)	0.013		
δSATI	0.556 (0.385–0.803)	0.002	0.513 (0.354–0.745)	<0.001	0.457 (0.232–0.901)	0.024	0.743 (0.363–1.520)	0.416

Abbreviations: CA 19‐9, carbohydrate antigen 19‐9; CCI, Charlson comorbidity index; CI, confidence interval; CTx, chemotherapy; ECOG, Eastern Cooperative Oncology Group; HR, hazard ratio; MA, muscle attenuation; SATI, subcutaneous adipose tissue index; SMI, skeletal muscle index; VATI, visceral adipose tissue index.

## Discussion

In this retrospective study, we found that initial body composition and its longitudinal changes were associated with the survival of patients with metastatic pancreatic cancer. Most body composition indices, except MA, changed the most in the first 2 months. Furthermore, the effect of initial body composition and its changes differed according to sex. In male patients, initial SMI and changes in SATI were significantly associated with OS, while in female patients, initial SATI was significant. Only initial MA, indicating muscle strength and quality, was a significant prognostic factor in both sexes. To our knowledge, this study is the first to analyse longitudinal changes in body composition for up to 6 months in pancreatic cancer. Based on our results, evaluation of both initial body composition and longitudinal changes in body composition using the deep learning‐based CT body segmentation technique could provide valuable information for predicting the prognosis of patients with metastatic pancreatic cancer.

Our results demonstrated that initial MA, which is closely related to skeletal muscle lipid content, was a significant prognostic factor in both sexes.^9^, ^19^ These findings are in agreement with previous studies, which reported that MA was associated with poor survival in patients with pancreatic or periampullary cancers.^20^, ^21^ In addition, a previous study reported an association between MA and incomplete adjuvant chemotherapy, which can result in poor OS.^22^ Additionally, initial SMI was a significant prognostic factor in male patients, which is in line with previous study results reporting an association between pancreatic cancer and sarcopenia in both curative and palliative treatment settings.^4^, ^16^, ^23^

Although many studies have investigated the prognostic value of sarcopenia in patients with pancreatic cancer, there has been no unified cut‐off of SMI for sarcopenia. Hence, previous studies used cut‐offs that were calculated from other studies^4^, ^16^ or directly inferred them within cohorts.^23^ In our study, we determined cut‐offs based on our cohort, which comprised patients with metastatic pancreatic cancer. The mean SMI values of our study were 43.5 cm^2^/m^2^ for males and 37.5 cm^2^/m^2^ for females. Although they were lower than those from early‐stage cohorts,^21^ they were comparable with those from advanced‐stage cohorts.^24^ When applying the cut‐off of low SMI in our cohort of 41.9 cm^2^/m^2^ for males and 39.2 cm^2^/m^2^ for females, the proportion of sarcopenia was 27.6% for males and 42.9% for females.

The restricted cubic spline curve of BMI was U‐shaped, which was consistent with other studies on both the western and asian populations.^25^, ^26^ However, the adipose tissue index showed a linear association, and the effects of VATI and SATI were opposite (*Figure* *S4*). To evaluate the individual effect of each body composition, we defined sex‐specific cut‐offs, instead of dividing several groups according to BMI. We found that initial SATI was a significant prognostic factor in female patients and that a decrease in SATI at 2‐month CT was a significant factor in male patients. Previous studies have shown heterogeneous results regarding the association between adipose tissue and prognosis of pancreatic cancer. In a retrospective cohort study by Ninomiya et al., there was no significant association between visceral adipose tissue area and OS in patients with resected pancreatic cancer.^27^ On the other hand, van Dijk et al. showed that low visceral adipose tissue area and high skeletal MA were associated with OS.^21^ In case of unresectable pancreatic cancer, Kays et al. reported a significant association between OS and changes of total fat area (SATI plus VATI) during chemotherapy.^28^ However, early changes in VATI or SATI were not associated with OS in a study by Salinas‐Miranda et al.^24^ Further validation with a larger population or multiple ethnicity is therefore needed.

Of note, in our study, the prognostic value of body composition parameters showed a difference between male and female patients. In male patients, both MA and SMI were significant prognostic factors, which is in agreement with previous studies.^23^, ^29^ On the other hand, SATI was a protective factor in female patients but did not show statistical significance in male patients. These differences according to sex imply sexual dimorphism in the cachexia phenotype, which has been verified in both preclinical and clinical studies.^30^, ^31^

In the Health ABC Study and the Baltimore Longitudinal Study on Aging, the decline in muscle strength was much greater than predicted by decline in muscle mass, especially in age over 85.^32^, ^33^ This finding suggests that muscle quality changes in later period of life. Similarly, MA, which is associated with muscle quality,^34^ decreased more prominently in 2–6 months than initial 2 months in our study. However, MA is not a direct indicator of muscle strength; longitudinal studies including measurement of muscle strength are needed.

The change in body composition results from the combined effect of nutrition, energy metabolism and inflammation in patients with cancer. The altered exocrine function of the pancreas is associated with sarcopenia and adipose tissue wasting.^35^ The imbalance between energy uptake and expenditure might contribute to cancer‐associated cachexia. Anorexia, nausea and vomiting might decrease energy intake regardless of chemotherapy, but the energy expenditure of cancer increases according to intrinsic metabolism and the rate of aerobic respiration.^36^ Inflammation also plays a major role in body composition changes. Numerous catabolic mediators and pro‐inflammatory cytokines such as activin, IL‐6 (interleukin‐6) and TWEAK (tumour necrosis factor‐like weak inducer of apoptosis) are associated with proteolysis, lipolysis and futile cycling in target organs, including skeletal muscle, cardiac muscle and adipose tissue.^37^, ^38^, ^39^ Therefore, evaluation of body composition changes can be helpful for the comprehensive assessment of disease and patient status, and management planning in patients with pancreatic cancer. However, further validation is needed.

This study had some limitations, mostly stemming from its retrospective design. First, some cases were excluded from analyses due to inappropriate CT or follow‐up loss. Although the number of these cases was small, it could have caused selection bias. Second, we assessed muscle mass without consideration of muscle strength or physical performance.^40^ Third, we lacked information on nutritional status and weight changes before diagnosis.^41^ Lastly, we analysed the effect of changes in body composition only with initial 2‐month period. In this cohort, patients with 6‐month CT scan showed significant differences in tumour size, CA 19‐9 level and performance scores when compared with those without 6‐month CT scan (*Table* *S1*). There was a possibility of a skewed result because this group might have longer survival times. In addition, it was difficult to analyse with Cox regression due to the heterogeneity of chemotherapy regimen, which included second‐line or third‐line treatment. Therefore, we analysed only with initial 2‐month CT scan. Further prospective studies that reflect muscle strength, physical performance and long‐term changes in body compositions are required.

In conclusion, in patients with metastatic pancreatic cancer, body composition changed the most during the first 2 months after starting chemotherapy, and the prognostic factors associated with OS differed between males and females. Initial and longitudinal changes of body composition are associated with OS of metastatic pancreatic cancer.

## Conflict of interest statement

The authors declare no conflicts of interest.

## Supporting information


**Table S1.** Comparison between groups according to presence of 6‐month CT scan.
**Figure S1.** Body composition analysis using CT image.
**Figure S2.** Maximally selected chi‐square test.
**Figure S3.** Cox proportional regression analysis with changes of body composition parameters in a 2‐month CT scan.
**Figure S4.** Restricted cubic spline curve.

**Data S1** Supporting Information.

## Data Availability

Individual participant data will not be shared.
