# Supplementary material for: Prognostic value of initial and longitudinal changes in body composition in metastatic pancreatic cancer
Source: J Cachexia Sarcopenia Muscle. 2024 Feb 8;15(2):735–45. doi: 10.1002/jcsm.13437 (PMC10995276; doi:10.1002/jcsm.13437)

**Supplementary Document 1** CT protocol

CT was performed at our institution (n=851; 73, 435, and 343 for initial, 2-month, and 6-month follow-up CT, respectively) or at outside hospitals (n=396; 383, 7, and 6 for initial, 2-month, 6-month follow-up CT, respectively) using various multi-detector row CT scanners and various protocols. Our institution’s pancreas CT protocol included unenhanced, early arterial, late arterial, and portal venous phase images. To obtain contrast-enhanced images, an intravenous contrast medium, iopromide (Ultravist 370; Schering, Berlin, Germany) was administrated at a dose of 1.5 mL/kg and a rate of 3–5 mL/sec followed by a 20-mL saline flush using an automatic power injector. Early arterial phase images were obtained 6–9 s after arriving at 100 Hounsfield unit (HU) in the descending aorta using the bolus tracking technique. Subsequently, late arterial (pancreatic parenchymal) and venous phase images were obtained at 35–50 and 60–70 s after starting the contrast injection, respectively. Axial images of portal venous phase had a section thickness of 2.5–3 mm or less in 224, 438, and 344 patients, and 4–5 mm in 232, 4, and 5 patients for initial, 2-month, and 6-month follow-up CT, respectively.

**Supplementary Table 1** Comparison between groups according to presence of 6-month CT scan

|  | With 6-month CT scan (N=349) | Without 6-month CT scan (N=107) | *P* value |
| --- | --- | --- | --- |
| Age | 60.9 ± 10.0 | 62.5 ± 10.1 | 0.143 |
| ECOG |  |  | 0.022 |
| 0 | 88 (25.2%) | 15 (14.0%) |  |
| ≥1 | 261 (74.8%) | 92 (86.0%) |  |
| Height | 163.4 ± 8.6 | 163.4 ± 8.5 | 0.980 |
| Weight | 60.5 ± 10.2 | 59.9 ± 10.7 | 0.639 |
| BMI | 22.6 ± 2.8 | 22.4 ± 3.2 | 0.529 |
| Type 2 diabetes | 121 (34.7%) | 37 (34.6%) | 1.000 |
| Dyslipidemia | 40 (11.5%) | 14 (13.1%) | 0.777 |
| Charlson comorbidity index |  |  | 0.063 |
| <10 | 310 (88.8%) | 87 (81.3%) |  |
| ≥10 | 39 (11.2%) | 20 (18.7%) |  |
| Tumor size | 4.0 ± 1.8 | 4.5 ± 2.0 | 0.026 |
| Tumor location |  |  | 0.488 |
| Head | 119 (34.1%) | 34 (31.8%) |  |
| Body | 121 (34.7%) | 33 (30.8%) |  |
| Tail | 109 (31.2%) | 40 (37.4%) |  |
| Peritoneal seeding | 99 (28.4%) | 34 (31.8%) | 0.577 |
| CA19-9 (U/mL) | 4139.4 ± 4904.8 | 5329.3 ± 5466.4 | 0.033 |
| Biliary obstruction | 58 (16.6%) | 18 (16.9%) | 1.000 |
| Duodenal obstruction | 10 ( 2.9%) | 3 ( 2.8%) | 1.000 |
| MA (HU) | 41.1 ± 9.1 | 41.0 ± 9.4 | 0.915 |
| SMI (cm^2^/m^2^) | 44.7 ± 7.5 | 43.9 ± 10.6 | 0.463 |
| VATI (cm^2^/m^2^) | 30.4 ± 19.8 | 31.5 ± 23.4 | 0.660 |
| SATI (cm^2^/m^2^) | 43.7 ± 22.8 | 41.4 ± 23.1 | 0.374 |

**Supplementary Figure 1** Body composition analysis using CT image

Portal venous phase (a) and mapped CT images (b) were obtained using DEEPCATCH at the same level of the L3 vertebral body. (b) Segmental axial CT image showing muscle area (red), visceral adipose tissue area (green), and subcutaneous adipose tissue area (yellow).

**
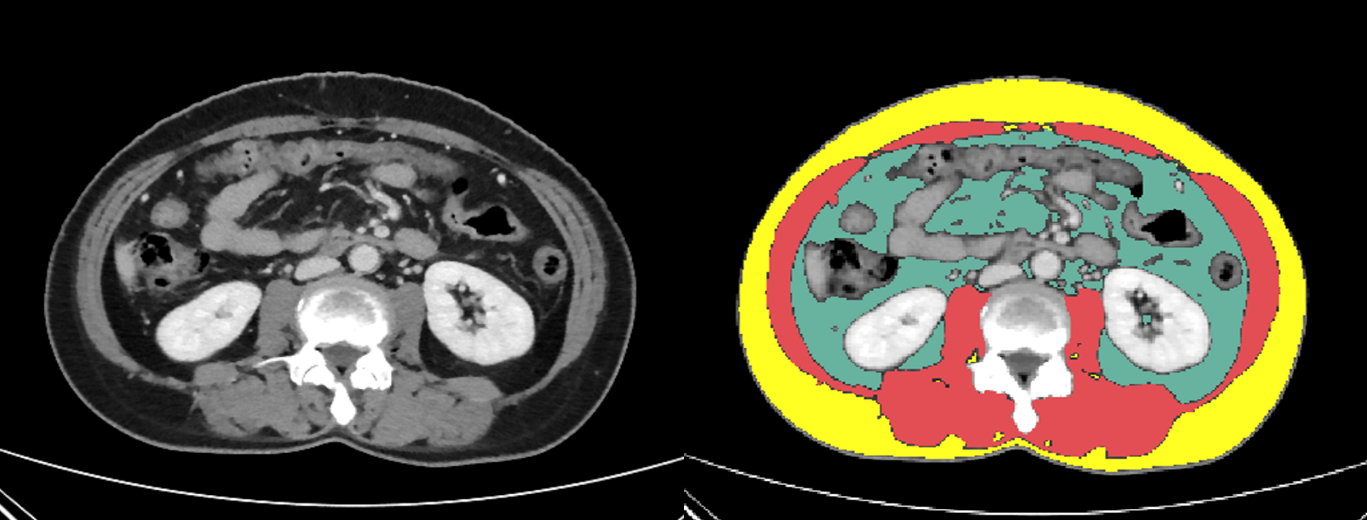
**

**Supplementary Figure 2** Maximally selected chi-square test

Sex-specific cut offs of each variable (a, Skeletal muscle index; b, Muscle attenuation; c, Visceral adipose tissue index; d, Subcutaneous adipose tissue index) were determined by maximally selected chi-square test.

a. Skeletal muscle index (SMI, cm^2^/m^2^)


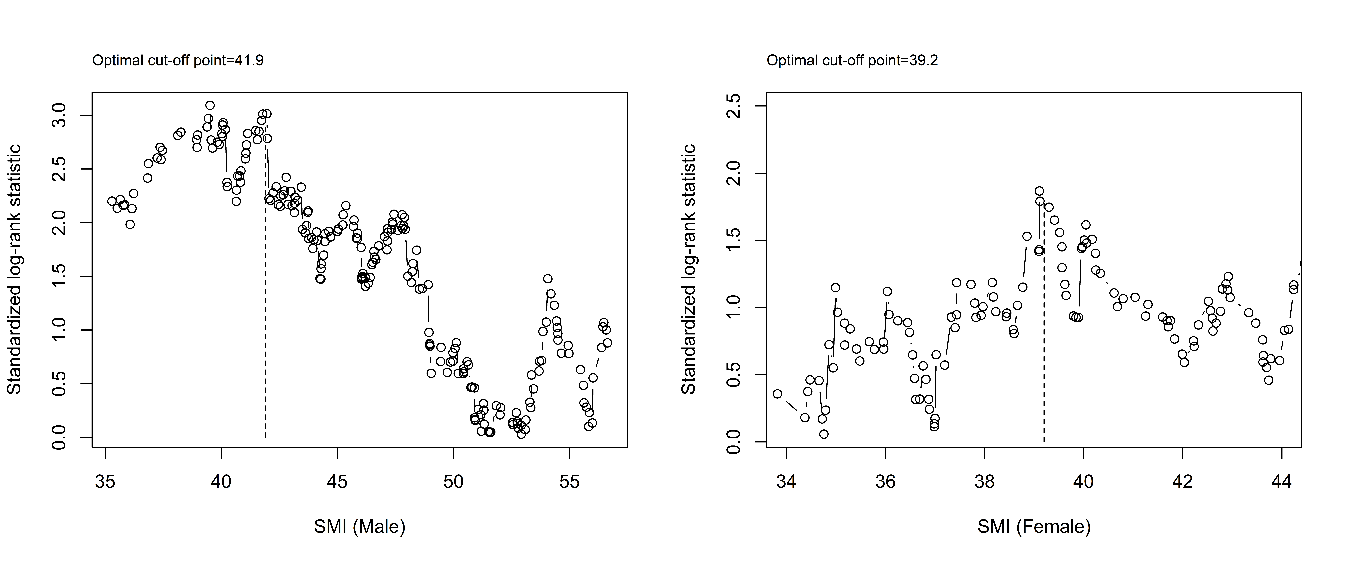


b. Muscle attenuation (MA, HU)


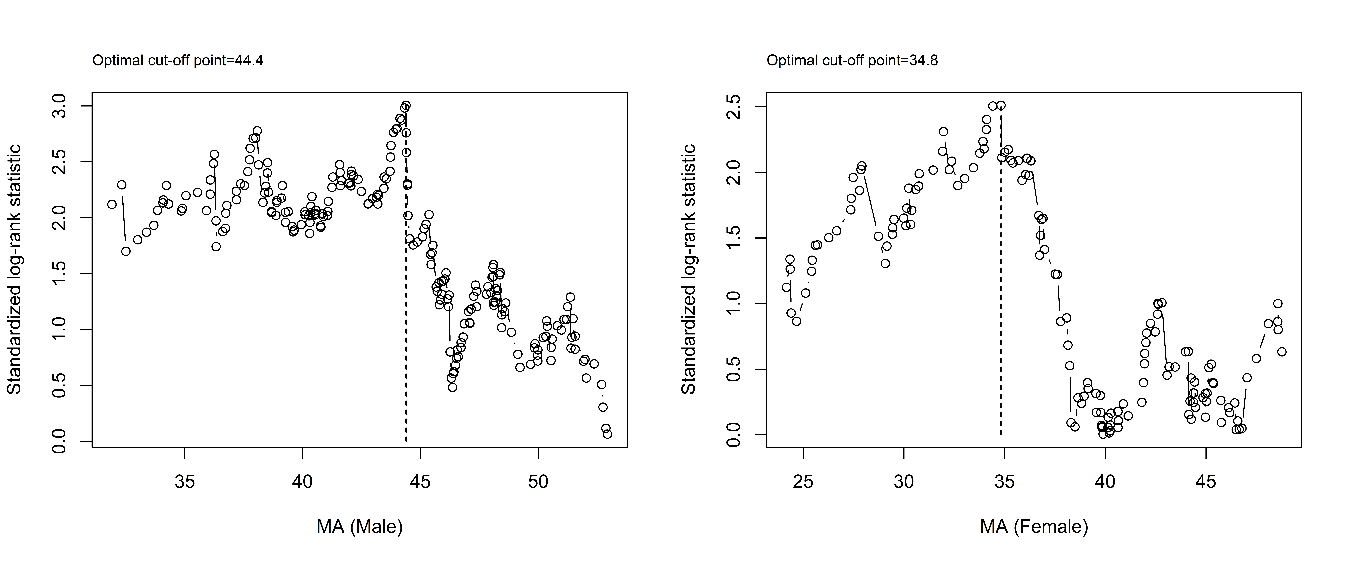


c. Visceral adipose tissue index (VATI, cm^2^/m^2^)


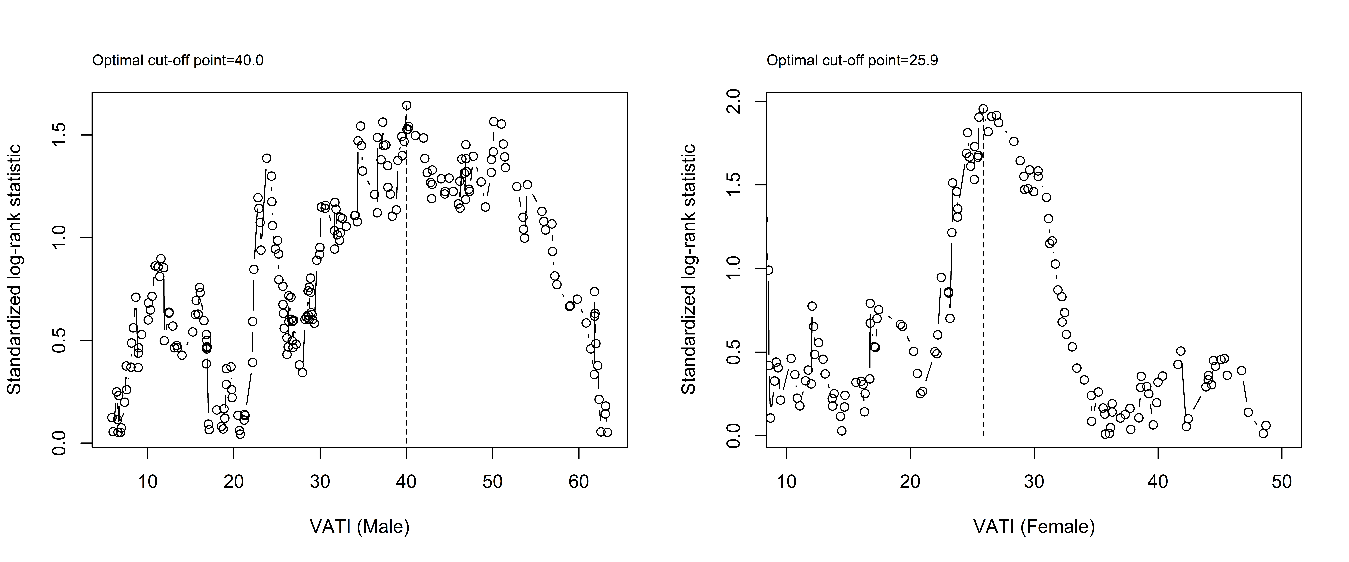


d. Subcutaneous adipose tissue index (SATI, cm^2^/m^2^)


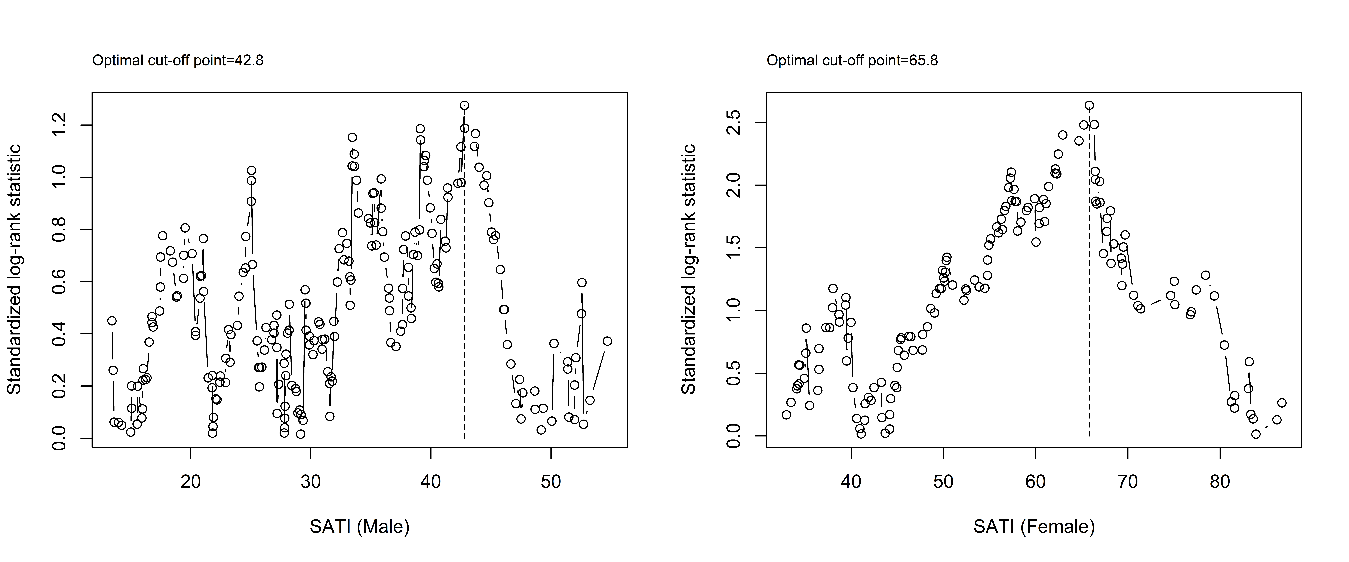


**Supplementary Figure 3** Cox proportional regression analysis with changes of body composition parameters in a 2-month CT scan

1. **
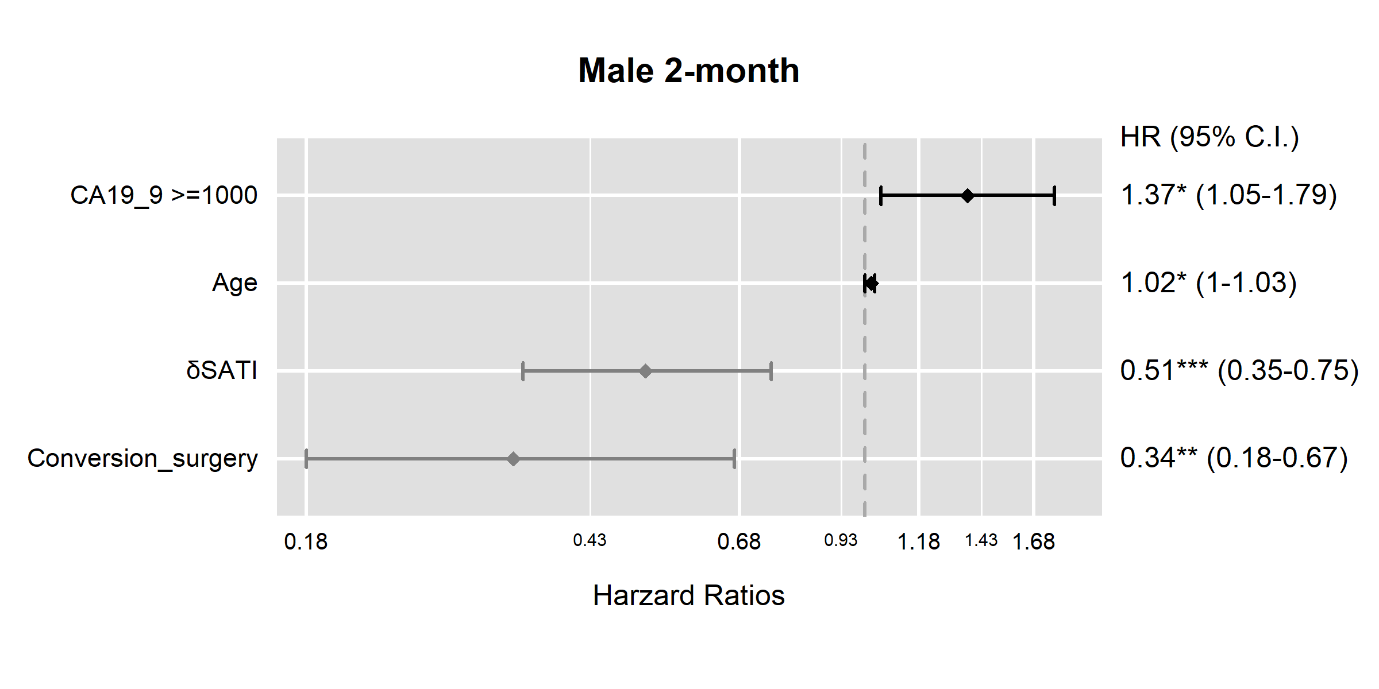
**Multi-variable Cox regression model for male
2.
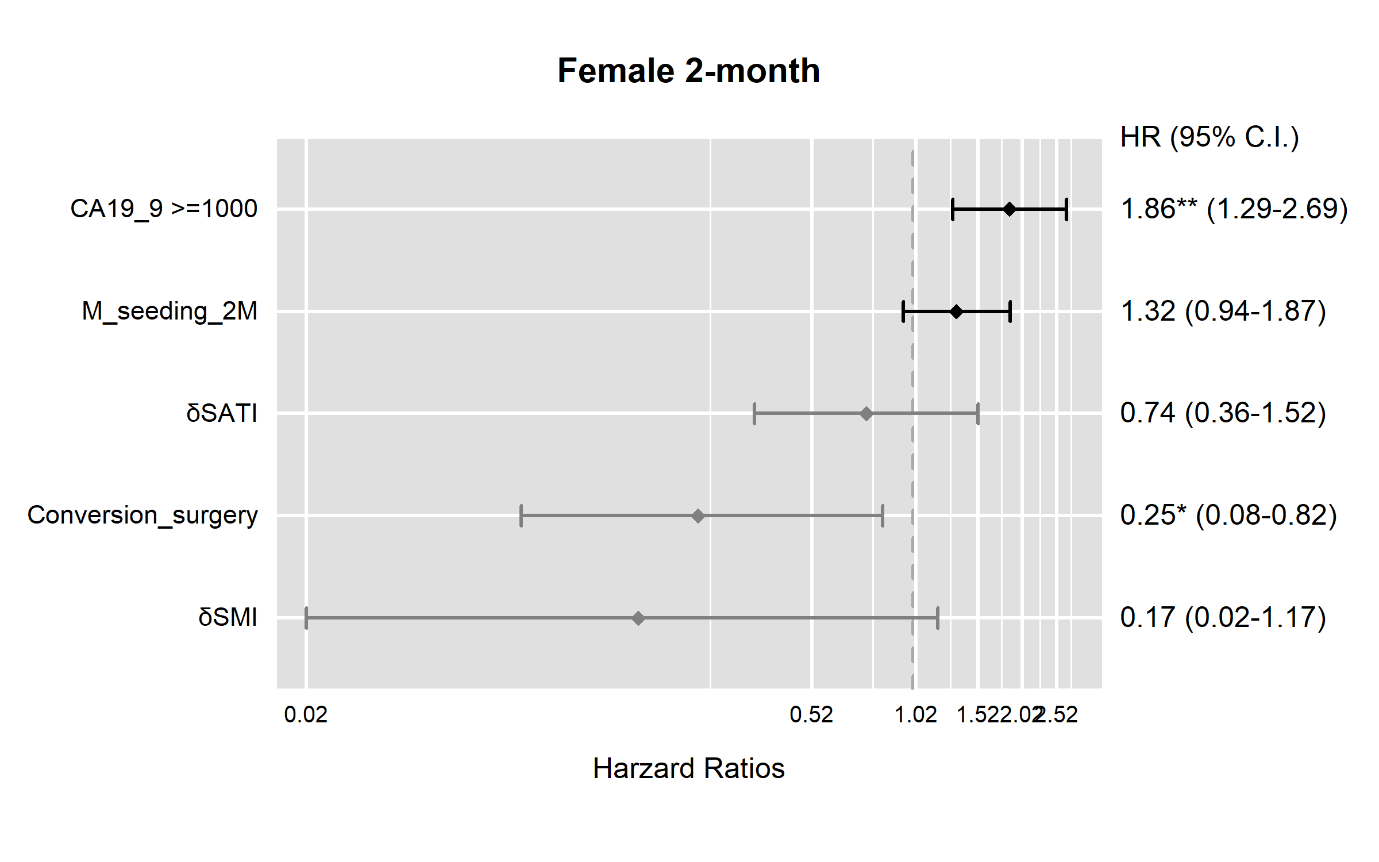
Multi-variable Cox regression model for female

**Supplementary Figure 4** Restricted cubic spline curve

a. Body mass index (BMI, kg/m^2^)


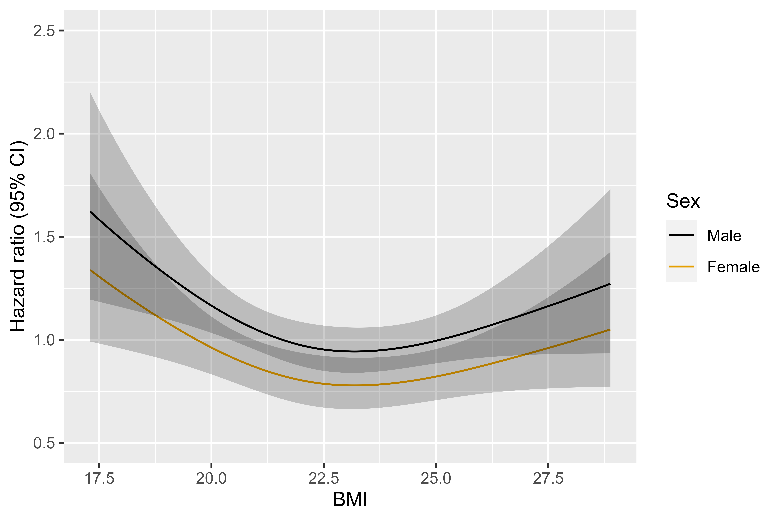


b. Visceral adipose tissue index (VATI, cm^2^/m^2^)


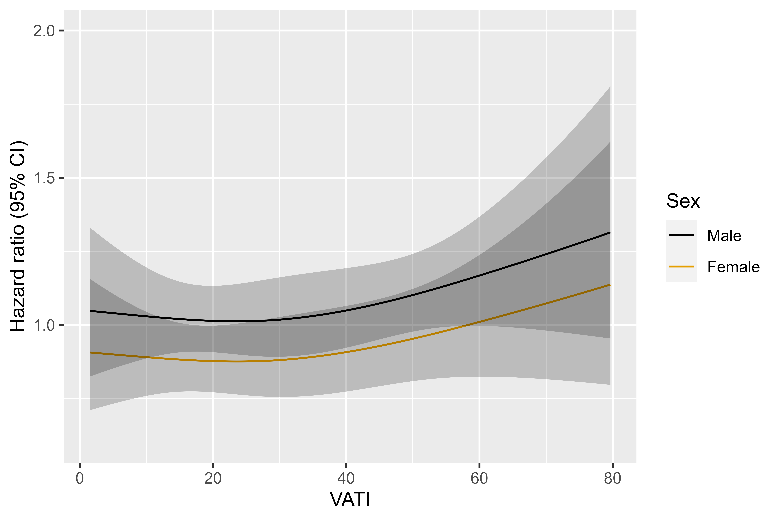


c. Subcutaneous adipose tissue index (SATI, cm^2^/m^2^)


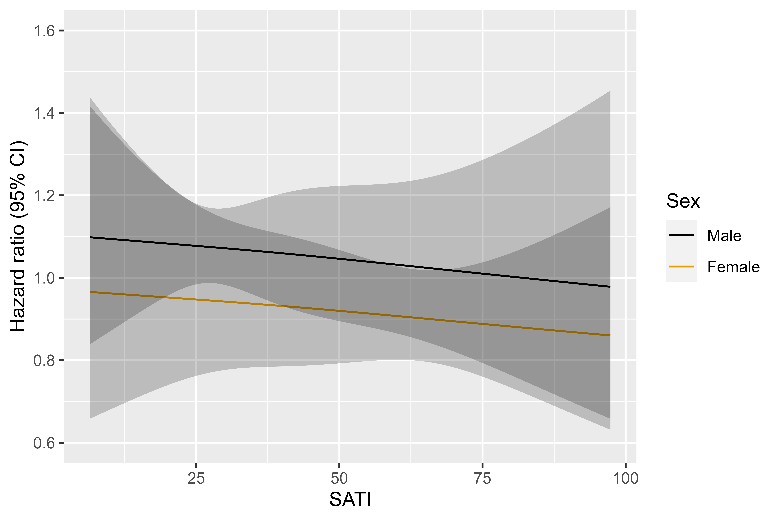

Supplement: Supplementary file 1 — Table S1. Comparison between groups according to presence of 6‐month CT scan. Figure S1. Body composition analysis using CT image. Figure S2. Maximally selected chi‐square test. Figure S3. Cox proportional regression analysis with changes of body composition parameters in a 2‐month CT scan. Figure S4. Restricted cubic spline curve. [file JCSM-15-735-s001.docx]
