# Supplementary material for: Prognostic value of initial and longitudinal changes in body composition in metastatic pancreatic cancer
Source: J Cachexia Sarcopenia Muscle. 2024 Feb 8;15(2):735–45. doi: 10.1002/jcsm.13437 (PMC10995276; doi:10.1002/jcsm.13437)
Supplement: Supplementary file 2 — Data S1 Supporting Information. [file JCSM-15-735-s002.pdf]

STROBE Statement—checklist of items that should be included in reports of observational studies

	Item No.	Recommendation	Page No.	Relevant text from manuscript
**Title and abstract**	1	(*a*) Indicate the study's design with a commonly used term in the title or the abstract	3	We retrospectively included 456 patients… from May 2011 to December 2019.
		(*b*) Provide in the abstract an informative and balanced summary of what was done and what was found	3	analyzed the association between body composition changes after chemotherapy and survival. Initial and longitudinal changes of body composition are associated with OS of metastatic pancreatic cancer.
**Introduction**				
Background/rationale	2	Explain the scientific background and rationale for the investigation being reported	5	Several studies have demonstrated … relatively less attention has been paid to other body compositions and metastatic pancreatic cancer.
Objectives	3	State specific objectives, including any prespecified hypotheses	6	Therefore, this study aimed to investigate the association of body composition and longitudinal changes after chemotherapy…
**Methods**				
Study design	4	Present key elements of study design early in the paper	7	This study was a retrospective cohort study…
Setting	5	Describe the setting, locations, and relevant dates, including periods of recruitment, exposure, follow-up, and data collection	7	histologically diagnosed with pancreatic ductal adenocarcinoma at our

				academic medical center between May 2011 and December 2019.
Participants	6	(*a*) *Cohort study*—Give the eligibility criteria, and the sources and methods of selection of participants. Describe methods of follow-up	7	Using our electronic medical record system… The following patients were included:… were excluded:…
		(*b*) *Cohort study*—For matched studies, give matching criteria and number of exposed and unexposed	N/A	
Variables	7	Clearly define all outcomes, exposures, predictors, potential confounders, and effect modifiers. Give diagnostic criteria, if applicable	7-8	Clinical data, including patient demographics, pretreatment height, pretreatment weight… Underlying diseases such as diabetes and dyslipidemia… Peritoneal seeding was determined…
Data sources/ measurement	8*	For each variable of interest, give sources of data and details of methods of assessment (measurement). Describe comparability of assessment methods if there is more than one group	9-10	Most factors were based on the date of the initial CT… they were determined based on each time point. The rate of interval change was calculated using the equations below…
Bias	9	Describe any efforts to address potential sources of bias	9	Survival analysis was performed according to sex since the body composition of each sex revealed different distributions.
Study size	10	Explain how the study size was arrived at	7	consecutive patients who were histologically diagnosed with … between May 2011 and December 2019.

Continued on next page

Quantitative variables	11	Explain how quantitative variables were handled in the analyses. If applicable, describe which groupings were chosen and why	10	The standard value of each body composition index according to sex was calculated with maximally selected rank statistics,
Statistical methods	12	(*a*) Describe all statistical methods, including those used to control for confounding	10	Variables with a P-value <0.2 in the univariable analysis were considered candidates for the multivariable model, which was determined by the bi-directional stepwise selection method.
		(*b*) Describe any methods used to examine subgroups and interactions	10	In the survival analysis of the 2-month time point, the Cox regression model was stratified by disease control state.
		(*c*) Explain how missing data were addressed	N/A	
		(*d*) *Cohort study*—If applicable, explain how loss to follow-up was addressed	N/A	
		(*e*) Describe any sensitivity analyses	N/A	
**Results**				
Participants	13*	(a) Report numbers of individuals at each stage of study—eg numbers potentially eligible, examined for eligibility, confirmed eligible, included in the study, completing follow-up, and analysed	11	Among 464 patients diagnosed with metastatic pancreatic cancer... For the analysis using longitudinal changes of body compositions, 99 patients without 2-month or 6-month follow-up CT and 8 patients who had inappropriate CT scan were excluded.
		(b) Give reasons for non-participation at each stage	11	The main reasons for treatment discontinuation were… Inappropriate CT scan was mainly due to…
		(c) Consider use of a flow diagram	11	Figure 1

Descriptive data	14*	(a) Give characteristics of study participants (eg demographic, clinical, social) and information on exposures and potential confounders	11	The baseline characteristics of the study population (n=456) are summarized in Table 1.
		(b) Indicate number of participants with missing data for each variable of interest	11	There were no missing values for all variables…
		(c) *Cohort study*—Summarise follow-up time (eg, average and total amount)	12	The median follow-up duration was 11.7 months (IQR 6.7-20.2).
Outcome data	15*	*Cohort study*—Report numbers of outcome events or summary measures over time	12	Among 456 patents, there were 452 deaths during follow up. The survival rate was 49.3% (95% confidence interval [CI], 44.9-54.2%) at 1 year, 9.0% (95% CI, 6.6-12.2%) and 3.7% (95% CI, 2.0-6.8%) at 5 year.
Main results	16	(*a*) Give unadjusted estimates and, if applicable, confounder-adjusted estimates and their precision (eg, 95% confidence interval). Make clear which confounders were adjusted for and why they were included	13	In the univariable and multivariable Cox regression analysis of male (Table 2) and female patients (Table 3)…
		(*b*) Report category boundaries when continuous variables were categorized	12	optimal cut-off by sex was calculated using maximally selected rank statistics (MA: 44.4 HU for males and 34.8 HU for females; SMI: 41.9 cm2/m2 for males and 39.2 cm2/m2 for females; VATI: 40.0 cm2/m2 for males and 25.9 cm2/m2 for females; SATI: 42.8 cm2/m2 for males and 65.8 cm2/m2 for females)
		(*c*) If relevant, consider translating estimates of relative risk into absolute risk for a meaningful time period	N/A	

Continued on next page

Other analyses	17	Report other analyses done—eg analyses of subgroups and interactions, and sensitivity analyses	12	Longitudinal changes in body composition parameters were assessed in a subset of patients (n=349) who had initial, 2-month, and 6-month CT.
**Discussion**				
Key results	18	Summarise key results with reference to study objectives	15	In this retrospective study, we found that initial body composition and its longitudinal changes were associated with the survival of patients with metastatic pancreatic cancer.
Limitations	19	Discuss limitations of the study, taking into account sources of potential bias or imprecision. Discuss both direction and magnitude of any potential bias	18	This study had some limitations, mostly stemming from its retrospective design.
Interpretation	20	Give a cautious overall interpretation of results considering objectives, limitations, multiplicity of analyses, results from similar studies, and other relevant evidence	15	Our results demonstrated that initial MA, which is closely related to skeletal muscle lipid content, was a significant prognostic factor in both sexes…
Generalisability	21	Discuss the generalisability (external validity) of the study results	17	Therefore, evaluation of body composition changes can be helpful for the comprehensive assessment of disease and patient status, and management planning in patients with pancreatic cancer.
**Other information**				
Funding	22	Give the source of funding and the role of the funders for the present study and, if applicable, for the original study on which the present article is based	N/A	

*Give information separately for cases and controls in case-control studies and, if applicable, for exposed and unexposed groups in cohort and cross-sectional studies.

Note: An Explanation and Elaboration article discusses each checklist item and gives methodological background and published examples of transparent reporting. The STROBE checklist is best used in conjunction with this article (freely available on the Web sites of PLoS Medicine at http://www.plosmedicine.org/, Annals of Internal Medicine at http://www.annals.org/, and Epidemiology at http://www.epidem.com/). Information on the STROBE Initiative is available at www.strobe-statement.org.
